# Editorial: Molecular and Cellular Crosstalk on Neuronal Functionality and Regulation, From Development to Pathology

**DOI:** 10.3389/fnmol.2022.966423

**Published:** 2022-06-30

**Authors:** Inmaculada Segura, Joaquim Egea

**Affiliations:** ^1^Max Planck Institute for Biological Intelligence (in Foundation), Planegg, Germany; ^2^Department of Cellular Physiology, Biomedical Center Munich, Ludwig Maximilian University, Planegg, Germany; ^3^Departament de Ciències Mèdiques Bàsiques, Universitat de Lleida, Lleida, Spain; ^4^Biomedical Research Institute of Lleida (IRBLLEIDA), Lleida, Spain; ^5^Serra Húnter Programme, Generalitat de Catalunya, Barcelona, Spain

**Keywords:** neuronal development, neuromodulation, neuronal maturation, regulation–physiological, connectivity

The development and functions of the nervous system are precisely guided and controlled by the crosstalk of molecular and cellular processes that are often revealed during pathological conditions, as defective molecular signaling and cellular interactions lie behind their cause or course. This Research Topic combines several reviews and original research articles highlighting the relevance of some of these processes, studied in different model systems, to broaden our understanding of the function of the nervous system.

Proteins and RNAs are key players of the cellular systems. Protein homeostasis, i.e., proteostasis, controls the quality and the appropriated amount of functional proteins within the cell (Giandomenico et al., 2022). Defective proteostasis has been linked to several disorders, including neurodegenerative diseases or behavior alterations. Therefore, as reviewed by Dudanova, the development of biosensors to assess protein quality is a promising approach to understand pathological mechanisms and to test possible therapies in the aforementioned conditions.

Among the proteins, the transcription factors play a central role during development due to their capacity to control gene expression. These genetic programs underlie several important processes, such as cellular proliferation, migration, or differentiation, which are essential, for instance, during nervous system development and function (Jain and Zipursky, 2022). The review by Tocco et al. summarizes the molecular and neurodevelopmental defects observed in a recently described neurodevelopmental monogenic syndrome, the Bosch-Boonstra-Schaaf Optic Atrophy Syndrome (BBSOAS). BBSOAS is characterized by the haploinsufficiency of the transcription regulator NR2F1 (previously known as COUP-TFI), which results in intellectual disability, visual impairment, epilepsy and autistic traits.

In the case of the RNAs, it has become evident in the recent years that RNAs display complex biological activities, e.g., messenger RNAs (mRNAs) are more than mere “photocopies” of the DNA to be translated into proteins or long non-coding RNAs (Lnc-RNAs) are not just junk biomolecules (Aprea and Calegari, 2015; Sonneveld et al., 2020). The reviews by Landínez-Macías and Urwyler and by Policarpo et al. summarize recent insights in either mRNA metabolism, with special emphasis on the RNA-binding protein Musashi, or in Lnc-RNAs, respectively, during neuronal development and function, from neural stem cell maintenance to synapse specification and wiring. They also explore their link to neurological diseases.

Neurons are very polarized cells whose dendrites and axons excess in length the size of the somas. Regulation of cytoskeleton dynamics plays a key function in polarity, size and connectivity which are essential for proper neuronal function in integrated circuits (Schelski and Bradke, 2017). As reviewed by Sánchez-Huertas and Herrera, the regulation of microtubule assembly, stability and its interaction with the actin network are processes particularly important at the growth cone of the developing axons, supporting axonal pathfinding and proper connectivity. But microtubules also play an important function as “railways” for motor proteins supporting the bidirectional trafficking of vesicles and organelles along the axons (Cason and Holzbaur, 2022). The review by Markworth et al. summarizes the trafficking defects that have been identified as the cause of the Charcot-Marie-Tooth disease, a neurodegenerative disease of the peripheral nervous system that cause axonal degeneration and neuronal loss.

Neurons are integrated in functional circuits that are mainly developed during embryonal and larval/infant stages and consolidated and refined during the adulthood. In that context, the study of small animals such as fish or rodents are of great value for the understanding of neuron connectivity. The work by Perez-García et al. demonstrates how the majority of the electrophysiological properties of the main effector neurons in the rat motor cortex, the layer V pyramidal neurons, are refined postnatally and are not defined until adulthood's onset. Another important postnatal refining process of neuronal circuits is that non-integrated cells are eliminated by physiological apoptosis (Wong and Marín, 2019). The work by Wang et al. warns about the use of the anesthetic sevoflurane, widely used in pediatrics, as it enhances this physiological apoptotic wave in the somatosensory cortex in mice. In the adulthood, neuromodulatory circuits allow adaptation of the behavior of the animals to their ever-changing needs, as for example during feeding behavior. The main neuromodulatory circuits are conserved among species. The review by Corradi and Filosa highlights the use of the translucent larva of the zebrafish, which facilitates *in vivo* imaging, in combination with appropriate behavioral tests and transgenesis to decipher the mechanisms of circuitry connection and behavioral modulation. In mammals, feeding behavior is regulated by neurotransmitters and neuropeptides. The review by Chen et al. focuses on the mechanisms of the glucagon-like peptide-1 (GLP-1) to regulate food uptake and the activation of its receptor (GLP-1R) in several brain regions.

An alternative and accessible model system for the study of the central nervous system (CNS) is the retina. This internal layer of the eye is originated from the diencephalon and remains connected to the brain by the optic nerve, that projects to different brain areas. These projection areas include the thalamus and other accessory nuclei. Among them, the inputs to the medial terminal nucleus (MTN) in the ventral midbrain are necessary for reflex eye movements and retinal image stabilization during vision (Yonehara et al., 2009). Within the retina, photoreceptors, bipolar neurons, and retinal ganglion cells (RGCs, forming the optic nerve) are hierarchically organized in structural layers that are well-vascularized. The work by Huang et al. describes the neuroprotective effect of 7,8-dihydroxyflavone (DHF) in the immature retina of P7 rats against hypoxic-ischemic injury, a major cause of acquired visual impairment in children from developing countries. DHF acts as a synthetic TrkB agonist independent of BDNF that activates Artemin upregulation and reduces gliosis in the insulted immature retina. In this Research Topic, the work by Ruff et al. uses a new inducible genetic FLRT3-CreERT2 knock-in mouse to identify a new subpopulation of RGCs with direction selective properties. These RGCs express the transmembrane protein FLRT3 and their axons specifically project to the MTN. FLRTs are multifunctional transmembrane proteins with important functions during nervous system development (Peregrina and Del Toro, 2020). One additional member of the FLRT family, the FLRT2, is analyzed in the work by Li et al. FLRT2 expression is followed during both, normal brain and spinal cord postnatal development and adulthood and during spinal cord injury. This work suggests that FLRT2 might be necessary for the formation of the glial component of the injury scar at the lesion region.

Finally, in the search for therapeutic approaches to treat neuronal dysfunctions, the work by Zirotti Rosenberg et al. analyses the effect of the synthetic exogenous cannabinoid WIN 55212-2 on behavioral convulsions induced by pentylenetetrazol (PTZ) in young rats, comparing the response of males and females. Studying sex as a biological variable is of great interest nowadays as it will help to reduce the gap in the knowledge of the health between genders.

## Author Contributions

All authors listed have made a substantial, direct, and intellectual contribution to the work and approved it for publication.

## Conflict of Interest

The authors declare that the research was conducted in the absence of any commercial or financial relationships that could be construed as a potential conflict of interest.

## Publisher's Note

All claims expressed in this article are solely those of the authors and do not necessarily represent those of their affiliated organizations, or those of the publisher, the editors and the reviewers. Any product that may be evaluated in this article, or claim that may be made by its manufacturer, is not guaranteed or endorsed by the publisher.
